# Non-Stimulatory pMHC Enhance CD8 T Cell Effector Functions by Recruiting Coreceptor-Bound Lck

**DOI:** 10.3389/fimmu.2021.721722

**Published:** 2021-10-11

**Authors:** Xiang Zhao, Liang-Zhe Wu, Esther K. Y. Ng, Kerisa W. S. Leow, Qianru Wei, Nicholas R. J. Gascoigne, Joanna Brzostek

**Affiliations:** ^1^Immunology Translational Research Programme, Yong Loo Lin School of Medicine, National University of Singapore, Singapore, Singapore; ^2^Department of Microbiology and Immunology, Yong Loo Lin School of Medicine, National University of Singapore, Singapore, Singapore

**Keywords:** co-agonism, Lck, T cell receptor, non-stimulatory peptide MHC, T cell signaling, T cell effector functions, AKT pathway

## Abstract

Under physiological conditions, CD8^+^ T cells need to recognize low numbers of antigenic pMHC class I complexes in the presence of a surplus of non-stimulatory, self pMHC class I on the surface of the APC. Non-stimulatory pMHC have been shown to enhance CD8^+^ T cell responses to low amounts of antigenic pMHC, in a phenomenon called co-agonism, but the physiological significance and molecular mechanism of this phenomenon are still poorly understood. Our data show that co-agonist pMHC class I complexes recruit CD8-bound Lck to the immune synapse to modulate CD8^+^ T cell signaling pathways, resulting in enhanced CD8^+^ T cell effector functions and proliferation, both *in vitro* and *in vivo*. Moreover, co-agonism can boost T cell proliferation through an extrinsic mechanism, with co-agonism primed CD8^+^ T cells enhancing Akt pathway activation and proliferation in neighboring CD8^+^ T cells primed with low amounts of antigen.

## Introduction

T cell receptor (TCR) on CD8^+^ T cells recognizes MHC class I (MHC-I) molecules presenting 8-11 amino acid-long peptides. These peptides are normally derived from proteolytic cleavage of proteins synthesized in a cell that expresses the MHC. In the absence of infection or antigenic mutations, MHC-I molecules exclusively present a repertoire of endogenous peptides. These self-peptide-MHC (pMHC) molecules do not normally induce T cell activation, as T cell clones with high reactivity against self pMHC are eliminated during the negative selection step of T cell development (1). However, a low degree of TCR reactivity against self pMHC is required for positive selection during thymocyte development, as well as for peripheral T cell survival and responsiveness (2, 3). Cellular production of non-self proteins, such as viral proteins, or of mutated self proteins, such as in tumors, results in presentation of peptides that can be recognized by high affinity TCRs. These antigenic peptides induce CD8^+^ T cell activation, leading to T cell effector functions and elimination of target cells bearing the antigenic peptides. However, the antigenic pMHC on infected or malignant cells are presented in the context of a large excess of non-stimulatory, self pMHC. There is strong evidence showing that these non-stimulatory pMHC can enhance T cell responses to antigenic pMHC, by a process termed co-agonism (4–6).

Co-agonism has been observed during mouse CD8^+^ T cell responses to TAP deficient antigen presenting cells (APCs) pulsed with varying amounts of agonist and non-stimulatory peptides (6–8). Co-agonism was also observed in mouse and human CD8^+^ T cells responding to engineered xenogeneic APCs presenting low density of agonist pMHC-I expressed in single chain (sc) format, in the presence or absence of sc non-stimulatory pMHC (6, 9). Moreover, multimers consisting of low numbers of agonist pMHC and non-stimulatory pMHC can co-operatively induce mouse and human CD8^+^ T cell activation (10–12). Dimers of pMHC consisting of a positively or negatively selecting pMHC and a non-stimulatory pMHC can mediate positive or negative selection of OT-I thymocytes, respectively (13). Co-agonism in CD8^+^ T cells is dependent on the CD8 coreceptor, rather than TCR, being able to bind to non-stimulatory pMHC class I complexes (6, 11). Most experiments investigating co-agonism focused on short-term T cell activation, such as Ca^2+^ flux and CD69 upregulation, and it is not yet known how co-agonism contributes to T cell signaling, proliferation, and development of effector functions. Co-agonism has also been implicated in CD4+ T cell responses. Non-stimulatory MHC class II were shown to accumulate at the immune synapse (14) and to augment CD4+ T cell activation (15, 16). However, the molecular mechanism of co-agonism in CD4+ T cell differs from that of CD8+ T cells, as it requires TCR, but not CD4, binding to co-agonist pMHC (17).

A major implication of MHC-I co-agonism is that CD8^+^ T cell activation is controlled, not only by the amount of agonist pMHC complexes, but also by the total MHC- surface density. Total cell surface MHC-I expression is upregulated during infection, in response to IFN-γ, and is frequently downregulated during viral infections and tumorigenesis. Herpesviruses, retroviruses and adenoviruses encode proteins that interfere with MHC-I cell surface expression by targeting different steps in the MHC-I presentation pathway (18). In an *in vivo* mouse model, this downregulation of MHC-I reduces CD8^+^ T cell effector functions (19). Critically, an increase in total cell surface density of HLA-C in HIV patients correlates with better control of HIV infection (20). Downregulation of pMHC-I surface expression is observed in multiple tumor types, including melanoma, colorectal, gastric and ovarian cancers (21). It constitutes a significant challenge for development of T cell-mediated tumor immunotherapies (22).

Under physiological conditions, CD8^+^ T cells can recognize low numbers of antigenic pMHC-I complexes in the presence of a large surplus of non-stimulatory, self pMHC-I on the APC cell surface (23–25). Therefore, co-agonism is likely to be a dominant modality of T cell recognition during responses against pathogens and tumors. We therefore investigated how co-agonism modulates T cell effector functions, proliferation, and signaling pathways at both single cell and population levels. As previous work from our lab has shown that co-agonism is stronger in naïve CD8^+^ T cells than in effector cytotoxic T lymphocytes (CTLs) (8), we focused on the role of co-agonism during priming of *ex vivo* CD8^+^ T cells. We show here that co-agonism enhances CD8^+^ T effector differentiation, as well as cell proliferation *in vitro* and *in vivo* during bacterial infection. Moreover, co-agonism boosts T cell proliferation through both cell-intrinsic and extrinsic mechanisms, with co-agonism primed CD8^+^ T cells enhancing PI3K-Akt pathway signaling and proliferation in neighboring CD8^+^ T cells primed with low amounts of antigen. Co-agonism increases Akt pathway activation, ERK phosphorylation and NFAT nuclear translocation. Co-agonist pMHC complexes have been shown to recruit CD8 coreceptor and active Lck to the immune synapse (7, 9). We show here that non-stimulatory pMHC-I complexes recruit CD8-bound Lck to the immune synapse, even in the absence of agonist pMHC-I. These results indicate that co-agonist pMHC-I recruit CD8-bound Lck to modulate T cell signaling pathways, resulting in enhanced CD8^+^ T cell effector functions and proliferation.

## Results

### Co-Agonism Enhances CD8^+^ T Cell Cytokine and Transcription Factor Expression

Recognition of non-stimulatory pMHC-I complexes has been shown to enhance early activation events in mouse and human CD8^+^ T cells (6–9). We sought to determine if co-agonist pMHC-I complexes can enhance cytokine production in OT-I CD8^+^ T cells, and expression of molecules implicated in CD8^+^ T cell effector differentiation. We used the T_REX_ CHO system (6, 26) as APCs, which allows presentation of small amounts of agonist OVA peptide on H-2K^b^ in a single chain format (scK^b^OVA) (27) in the presence or absence of non-stimulatory VSV on H-2K^b^, also in single chain format (scK^b^VSV). As T_REX_ CHO cells do not express any mouse MHC-I molecules, use of this system allows us to compare T cell responses to low amount of agonist pMHC in the presence or absence of non-stimulatory pMHC. We refer to scK^b^VSV as co-agonist pMHC-I when it is co-expressed with agonist pMHC-I, but as non-stimulatory pMHC-I when it is present in the absence of agonist pMHC-I. scK^b^OVA was expressed under the control of tetracycline-inducible promoter, with very low, “leaky” expression of scK^b^OVA in the absence of tetracycline (OVA^low^ cells), and high expression of scK^b^OVA after addition of tetracycline (OVA^hi^ cells). scK^b^OVA cells were super-transfected with scK^b^VSV under control of constitutive promoter, resulting in simultaneous presentation of low amounts of agonist pMHC-I and high amounts of non-stimulatory pMHC-I (OVA^low^-VSV cells) (**Supplementary Figure 1A**). The untransfected T_REX_ CHO cells (T_REX_) and T_REX_ CHO constitutively expressing scK^b^VSV (VSV) were used as negative controls.

**Figure 1:**
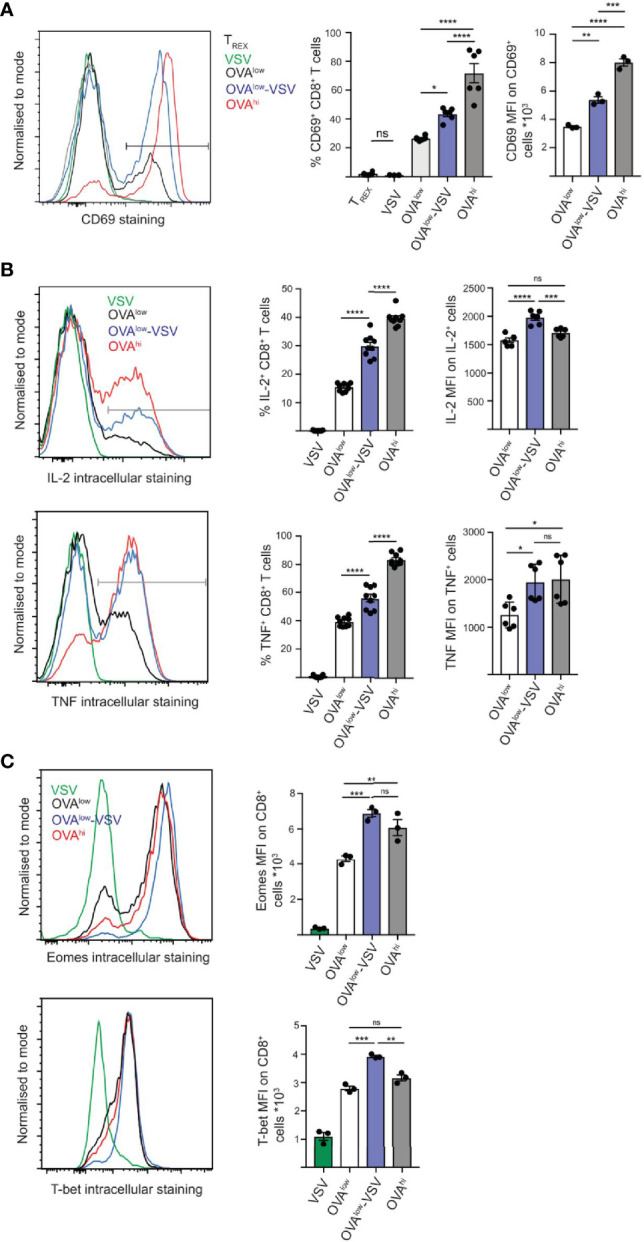
Co-agonism enhances CD8^+^ T cell activation and transcription factor expression. *Ex vivo* OT-I lymphocytes were stimulated with the indicated CHO APCs for 3h **(A)**, 5h **(B)** or 24h **(C)**, followed by surface staining to detect CD69 activation marker **(A)**, or intracellular staining to detect intracellular cytokines **(B)** or transcription factors **(C)** on CD8^+^ T cells. **(A)** Data from 6 mice from 2 independent experiments (%) or 3 mice from 1 experiment, representative of 2 independent experiments (MFI). **(B)** Data from 9 mice from 3 independent experiments (%) or 6 mice from 2 experiments (MFI), representative of 3 independent experiments. **(C)** Data from 3 mice from 1 experiment, representative of 3 independent experiments. Statistical significance was calculated using one-way ANOVA, with Tukey’s multiple comparisons test. *P < 0.05, **P < 0.01, ***P < 0.001, ****P < 0.0001, ns, P ≥ 0.05.

We confirmed our previous finding that presence of scK^b^VSV enhances OT-I CD8^+^ T cell responses to low amount of scK^b^OVA in this experimental system using CD69 upregulation after 3h stimulation as a readout (**Figure 1A**). CD69 is an early marker of T cell activation, and our time course analysis shows that 3h stimulation gives the optimal dynamic range, defined as the difference between CD8^+^ T cell response to OVA^low^ and OVA^hi^ cells (**Supplementary Figure 1B**). As expression of scK^b^VSV alone does not induce OT-I CD8^+^ T cell activation (**Figure 1A**), we used T_REX_ or VSV APCs as negative controls for T cell activation in the subsequent experiments. Critically, non-stimulatory pMHC-I enhanced T cell activation on both population (percentage of cells upregulating CD69) and single cell level (mean CD69 expression on CD69^+^ cells). This effect of co-agonist pMHC-I is apparent only for stimulation with low amounts of antigen, as stimulation with OVAhi-VSV cells resulted in comparable T cell activation as that with OVA^hi^ cells (**Supplementary Figure 1C**).

We then quantified cytokine production at the single cell level using intracellular staining and flow cytometry. Stimulation with OVA^low^-VSV resulted in a significantly higher proportion of CD8^+^ T cells producing IL-2 and TNF, as well as higher amounts of cytokines being produced at the single cell level, as compared to stimulation with OVA^low^ (**Figure 1B**). These results indicate that co-agonism can enhance T cell effector functions at both population and single cell levels. We then tested if this is correlated with enhanced expression of the T-box transcription factors, Eomes (Eomesodermin) and T-bet (T-box expressed in T-cells), with critical roles in regulation of T cell differentiation and effector functions (28, 29). To determine whether co-agonist pMHC can enhance the upregulation of Eomes and T-bet, *ex vivo* OT-I T cells were stimulated for 24h using the panel of CHO APCs. Expression of Eomes and T-bet was higher after stimulation with OVA^low^-VSV, as compared to OVA^low^ cells, indicating that co-agonism augments the magnitude of expression of T-box transcription factors in T cells responding to low amounts of antigenic pMHC (**Figure 1C**). Overall, our data show that co-agonism enhances cytokine production and expression of T-box transcription factors important for T cell effector functions and differentiation. Intriguingly, co-agonist pMHC can enhance single cell T cell responses to levels comparable to or higher than stimulation with high amount of agonist pMHC. This was apparent for cytokine production and the T-box transcription factor expression, suggesting that co-agonism-aided stimulation is uniquely efficient in supporting differentiation of effector CD8^+^ T cells.

### Co-Agonism Enhances CD8^+^ T Cell Proliferation *In Vitro* and *In Vivo*

**Figure 2:**
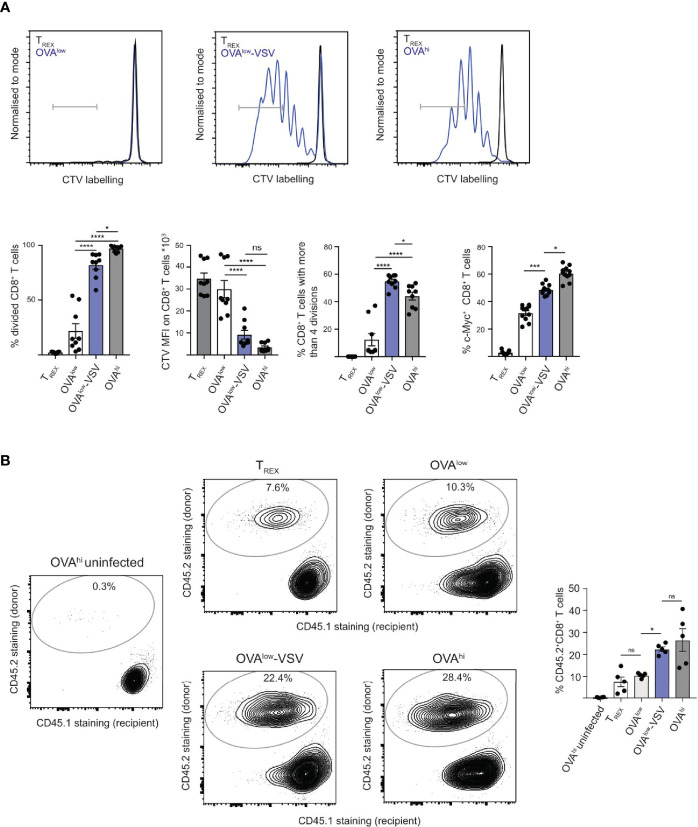
Co-agonism enhances CD8^+^ T cell proliferation *in vitro* and *in vivo*. **(A)** *Ex vivo* OT-I lymphocytes were labelled with CTV and stimulated with the indicated CHO APCs for 4h, followed by transfer of T cells into wells without APCs, and proliferation analysis 3 days post-transfer. For c-Myc expression analysis, unlabeled OT-I cells were stimulated with the CHO APCs for 4h, followed by fixation and staining. Data from 9 mice from 3 independent experiments, with representative flow cytometry flow plots shown. **(B)** *Ex vivo* OT-I lymphocytes (CD45.2^+^) were stimulated with the indicated CHO APCs for 4h, followed by adoptive transfer into CD45.1^+^ recipients. One day post-transfer, the recipient mice were infected with 10^4^ cfu LM-OVA, and the expansion of the donor-derived CD45.2^+^ CD8^+^ T cells was analyzed 4 days post-infection. Flow cytometry plots show CD45.1 and CD45.2 staining on the CD8^+^ T cell population, representative of 2 independent experiments. Data from 1 experiment with 5 recipient mice, representative of 2 independent experiments. Statistical significance was calculated using one-way ANOVA, with Tukey’s multiple comparisons test. *P < 0.05, ***P < 0.001, ****P < 0.0001, ns, P ≥ 0.05.

Activation induces T cell proliferation, which is critical for responses against pathogens *in vivo*. As co-agonism enhances T cell cytokine production and T-box transcription factor expression, we sought to determine whether co-agonism augments T cell proliferation under physiologically relevant stimulation conditions. Conventional *in vitro* proliferation assays use continuous 3 day antigen stimulation in the absence of exogenous cytokines. However, under physiological conditions *in vivo*, T cell priming occurs within the timeframe of hours, not days (30, 31), in the presence of homeostatic cytokines such as IL-7. Relatively short stimulation with antigen is sufficient to support CD8^+^ T cell commitment to antigen-independent proliferation *in vitro* (32). We therefore performed *in vitro* proliferation assays by co-culturing CTV-labelled OT-I CD8^+^ T cells with the CHO APC panel in the presence of low concentrations of IL-7 for 4h, followed by transfer of T cells into wells without APCs and subsequent culture in the presence of low concentrations of IL-7 for 3 days. In this experimental system, we observed no proliferation in response to T_REX_ used as a negative control (**Figure 2A**), thus confirming that neither T_REX_ APCs nor IL-7 at the concentration used can induce T cell proliferation (33). Stimulation with OVA^low^ cells resulted in approximately 20% cell division, but stimulation with OVA^low^-VSV or OVA^hi^ APCs induced more than 80% cell division (**Figure 2A**), indicating that co-agonism enhances T cell proliferation under physiologically relevant conditions *in vitro*. Co-agonism-aided T cell activation resulted in high dilution of the CTV signal, suggesting that activated cells underwent multiple rounds of division (**Figure 2A**). In agreement with this, co-agonism did not just enhance the proportion of cells undergoing division, but also augmented the number of divisions per cell, with less than 15% of cells undergoing at least 5 divisions after stimulation with OVA^low^ APC, but with approximately 50% of T cells undergoing at least 5 divisions after stimulation with OVA^low^-VSV or OVA^hi^ (**Figure 2A**). The transcriptional regulator c-Myc is critical for T cell proliferation (34), and the proportion of cells expressing c-Myc has been shown to correlate with TCR signal strength (35). We quantified c-Myc upregulation at 4h post-stimulation with the CHO APC panel, and discovered that stimulation with OVA^low^-VSV resulted in c-Myc expression in a higher proportion of CD8^+^ T cells than stimulation with OVA^low^ APCs (**Figure 2A**). Our results show that presence of co-agonist pMHC during CD8^+^ T cell priming increases c-Myc expression and proliferation *in vitro*.

As our data show that co-agonism enhances T cell proliferation *in vitro*, we investigated if co-agonism affects T cell expansion *in vivo*. 4h stimulation with agonist pMHC *in vitro* results in a very limited number of divisions after transfer into antigen-free recipient mice (36). However, we hypothesized that 4h priming *in vitro* will program CD8^+^ T cells for better proliferation *in vivo* upon re-encounter with the antigenic pMHC during infection. To test this prediction, we primed OT-I lymphocytes with the CHO APC panel *in vitro* for 4h, followed by adoptive transfer into congenic CD45.1 recipient mice. One day after the transfer, the mice were infected with *Listeria monocytogenes* expressing OVA (LM-OVA) (37). We analyzed proliferation of the transferred OT-I CD8^+^ T cells by quantifying the proportion of donor-derived CD45.2^+^ cells in the CD8^+^ T cell population at day 4 post-infection. Uninfected recipients that received OT-I T cells stimulated with OVA^hi^ APCs were used as controls to test whether *in vivo* OT-I proliferation requires LM-OVA infection (**Figure 2B**). Upon LM-OVA infection, we observed comparable *in vivo* proliferation of OT-I T cells primed with T_REX_ or OVA^low^ APCs. Critically, priming with OVA^low^-VSV resulted in higher proliferation than that with OVA^low^. Priming with OVA^hi^ resulted in proliferation comparable to priming with OVA^low^-VSV (**Figure 2B**). Our data thus show that co-agonism enhances T cell proliferation *in vitro* and *in vivo*, suggesting that non-stimulatory pMHC contribute to T cell expansion during responses to infections.

### Co-Agonism Enhances CD8^+^ T Cell Responses at Population Level Though Quorum Sensing

CD8^+^ T cell proliferation can be regulated at the population level though PI3K-Akt pathway-dependent integration of TCR and cytokine signals (38). Strongly activated CD8^+^ T cells secrete high amounts of IL-2, which can synergize with suboptimal TCR signal to drive proliferation of weakly stimulated cells (38, 39). As a result, proliferation of individual T cells can be influenced by the activation status of neighboring T cells, akin to quorum sensing in bacteria (40). We have observed that presence of co-agonist pMHC enhanced both the proportion of cells producing IL-2 and cytokine production on a per cell basis within the responding population (**Figure 1B**). Therefore, we hypothesized that under conditions of low antigen availability, enhanced IL-2 production by CD8^+^ T cells after co-agonism-aided activation will increase the response at the population level, through a quorum sensing mechanism. To test this hypothesis, we used CHO APCs to stimulate OT-I lymphocytes labelled with different proliferation dyes, followed by APC-free co-incubation of lymphocytes activated by different APCs.

**Figure 3:**
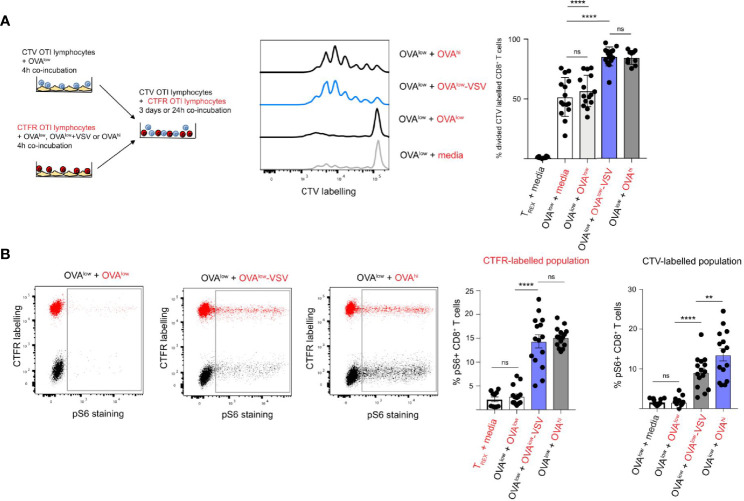
Co-agonism enhances CD8^+^ T cell quorum responses. **(A)** CTV or CTFR labelled OT-I lymphocytes were separately stimulated with the indicated CHO APCs for 4h, followed by co-culture of the differentially labelled population for 3 days in the absence of APCs. The representative flow cytometry plots show proliferative dye dilution in CTV-labelled CD8^+^ T cells primed with OVA^low^ APCs co-cultured with CTFR-labelled OT-I cells primed with the indicated CHO APCs (indicated in red). Data from 15 mice from 3 independent experiments. **(B)** CTV or CTFR labelled OT-I lymphocytes were separately stimulated with the indicated CHO APCs for 4h, followed by co-culture of the differentially labelled population for 20h in the absence of APCs. S6 phosphorylation on CD8^+^ T cells was assessed using intracellular staining. The representative flow cytometry plots show pS6 in co-cultured CTFR-labelled (CTFR^+^ population, indicated in red) and CTV-labelled (CTFR^–^ population, indicated in black) CD8^+^ T cells primed with the indicated APCs. The graphs show % of pS6^+^ CD8^+^ T cells from CTFR and CTV-labelled population, with the priming conditions for CTFR-labelled population indicated in red, and the priming conditions for CTV-labelled population in black. Data from 15 mice from 3 independent experiments. Statistical significance was calculated using one-way ANOVA, with Tukey’s multiple comparisons test. **P < 0.01, ****P < 0.0001, ns, P ≥ 0.05.

OT-I cells labelled with Cell Trace Violet (CTV) were stimulated with OVA^low^ APCs, whereas OT-I cells labelled with Cell Trace Far Red (CTFR) were stimulated with OVA^low^, OVA^low^-VSV or OVA^hi^ APCs (**Figure 3A**). After 4h of stimulation with CHO APCs, the CTV and CTFR-labelled OT-I cells were co-incubated for 3 days, followed by flow cytometry analysis of cell proliferation. As a control, CTV-labelled OT-I cells stimulated by OVA^low^ APCs were incubated with media alone, instead of co-incubation with CTFR-labelled OT-I cells. Stimulation with OVA^low^ APCs resulted in approximately 50% cell division in the CTV-labelled population, which was not affected by co-incubation with CTFR-labelled OT-I cells pre-activated with OVA^low^ APCs (**Figure 3A**). However, co-incubation with CTFR-labelled OT-I cells primed with OVA^hi^ APCs increased proliferation of CTV-labelled OT-I pre-activated with OVA^low^ APCs to more than 90% (**Figure 3A**). This indicates that proliferation of CD8^+^ T cells is strongly influenced by the activation status of the neighboring cells in this experimental system. Critically, presence of CTFR-labelled OT-I cells primed with OVA^low^-VSV enhanced proliferation of OVA^low^ primed CTV-labelled OT-I cells to more than 90% (**Figure 3A**), showing that co-agonism plays a role in T cell quorum sensing.

Studies using inhibitors have shown that the PI3K pathway is critical for integration of TCR and cytokine signals during CD8^+^ T cell quorum sensing (38). As we observed that the presence of co-agonism-primed OT-I T cells enhanced proliferation of OT-I T cells primed with low amounts of antigen, we sought to establish if co-agonism enhanced PI3K pathway signals in a cell-intrinsic and/or extrinsic manner. To answer this question, we quantified phosphorylation of S6 ribosomal protein (S6) in differentially labelled and primed CD8^+^ T cells after 24h of co-incubation. S6 is a downstream target of the PI3K pathway, as well as CARD11-BCL10-MALT1 and mTORC1 pathways (41, 42), and S6 phosphorylation in response to IL-2 was shown to depend on PI3K (38). We primed CTV-labelled OT-I cells with OVA^low^ APCs, and primed CTFR-labelled OT-I cells with OVA^low^, OVA^low^-VSV or OVA^hi^ APCs. After 24h of co-culture, we quantified the percentage of CD8^+^ T cells with pS6 signal in CTFR- and CTV-labelled cells to examine the cell-intrinsic and extrinsic effects of co-agonism, respectively. Priming with OVA^low^ APCs induced very low pS6 signal in CTFR-labelled cells, but priming with OVA^hi^ APCs induced pS6 signal in a sizeable proportion of CTFR-labelled cells (**Figure 3B**). Priming with OVA^low^-VSV APCs increased pS6 signal to that comparable to OVA^hi^ priming. These results show that co-agonism enhances PI3K pathway signaling in a cell-intrinsic manner. We then investigated whether co-agonism also increases PI3K pathway signals in a cell extrinsic manner, by analyzing pS6 in CTV-labelled cells primed with OVA^low^ APCs after co-incubation with CTFR-labelled cells primed with the different CHO APCs. Co-culture with CTFR-labelled T cells primed with low amount of antigen did not alter S6 phosphorylation in CTV-labelled T cells primed with low amount of antigen. However, the presence of CTFR^+^ T cells primed with high amount of antigen increased pS6 in CTV^+^ T cells primed with low amount of antigen (**Figure 3B**), showing that PI3K pathway signaling in CD8^+^ T cells can reflect the population-level activation status. Critically, co-culture with CTFR^+^ T cells primed with OVA^low^-VSV APCs increased pS6 signal in CTV^+^ CD8^+^ T cells primed with OVA^low^ APCs (**Figure 3B**), indicating that co-agonism has cell extrinsic effects on PI3K pathway activation. Our data show that co-agonism can enhance CD8^+^ T cell proliferation through cell extrinsic activation of the PI3K pathway.

### Co-Agonism Enhances CD8^+^ CTL Differentiation

**Figure 4:**
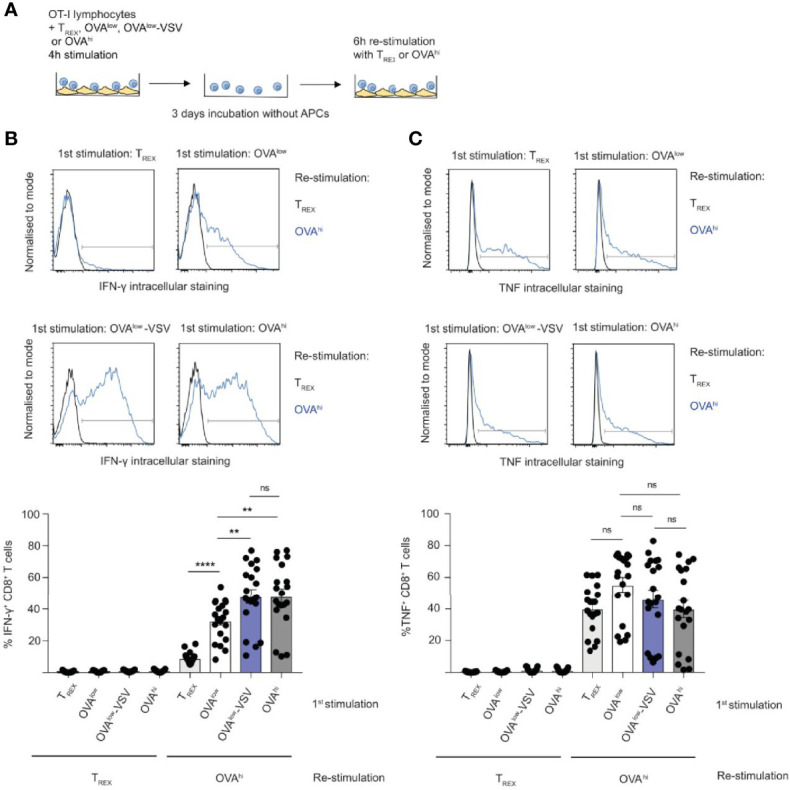
Co-agonism enhances CD8^+^ T cell effector differentiation. **(A)** *Ex vivo* OT-I lymphocytes were stimulated with the indicated CHO APCs for 4h, cultured for 3 days without APCs, followed by 6h re-stimulation with T_REX_ or OVA^hi^ APCs. IFN-γ **(B)** and TNF **(C)** production by CD8^+^ T cells was quantified using intracellular staining. Data from 19 mice from 4 independent experiments, representative flow cytometry plots are shown. Statistical significance was calculated using one-way ANOVA, with Tukey’s multiple comparisons test. **P < 0.01, ****P < 0.0001, ns, P ≥ 0.05.

Effective CD8^+^ T cell-mediated immune responses depend on cytotoxic T lymphocyte (CTL) differentiation from naïve CD8^+^ T cells after antigen recognition. Unlike naïve CD8^+^ T cells, CTLs can exert effector functions, such as IFN-γ production and cytotoxicity. We discovered that co-agonism increases expression of T-box transcription factors implicated in CTL differentiation and function (**Figure 1C**). Moreover, co-agonism enhances CD8^+^ T cell commitment to proliferation after 4h stimulation with APCs, and similar stimulation conditions were shown to be sufficient for CTL differentiation (32). We therefore asked if co-agonism can boost CD8^+^ T cell effector differentiation. To answer this question, we stimulated *ex vivo* OT-I lymphocytes for 4h with the CHO APC panel, followed by culture of the OT-I lymphocytes without APCs for 3 days (**Figure 4A**). CTL differentiation was then assessed by re-stimulating OT-I lymphocytes with OVA^hi^ CHO APCs, with untransfected T_REX_ cells used as a negative control. Stimulation with T_REX_ APCs did not induce CTL differentiation, as OT-I T cells stimulated with T_REX_ did not produce IFN-γ, a key CTL effector cytokine, upon re-stimulation with OVA^hi^ APCs (**Figure 4B**). This specifically reflects a lack of CTL differentiation, rather than impaired activation or viability, as T_REX_-stimulated OT-I CD8^+^ T cells produced substantial amounts of TNF upon OVA^hi^ re-stimulation (**Figure 4C**). Stimulation with OVA^low^ APCs was sufficient to drive CTL differentiation in a substantial proportion of OT-I CD8^+^ T cells, as approximately 40% of CD8^+^ T cells produced IFN-γ after re-stimulation with OVA^hi^ APCs (**Figure 4B**). Co-agonist pMHC-I augmented CTL differentiation, as stimulation with OVA^low^-VSV resulted in IFN-γ production by approximately 60% of OT-I CD8^+^ T cells after OVA^hi^ re-stimulation; this was comparable to IFN-γ production by OT-I T cells primed with OVA^hi^ APCs (**Figure 4B**). Unlike that of IFN-γ, TNF production was not dependent on the initial stimulation, with comparable TNF production upon OVA^hi^ re-stimulation by OT-I T cells stimulated by T_REX_, OVA^low^, OVA^low^-VSV and OVA^hi^ (**Figure 4C**). Moreover, stimulation with OVA^low^-VSV augmented CTL cytotoxicity, measured using cell surface staining of CD107a degranulation marker (**Supplementary Figure 1D**). Our results show that co-agonism can boost CTL differentiation.

### Co-Agonism Enhances ERK Phosphorylation and NFAT Nuclear Translocation

**Figure 5:**
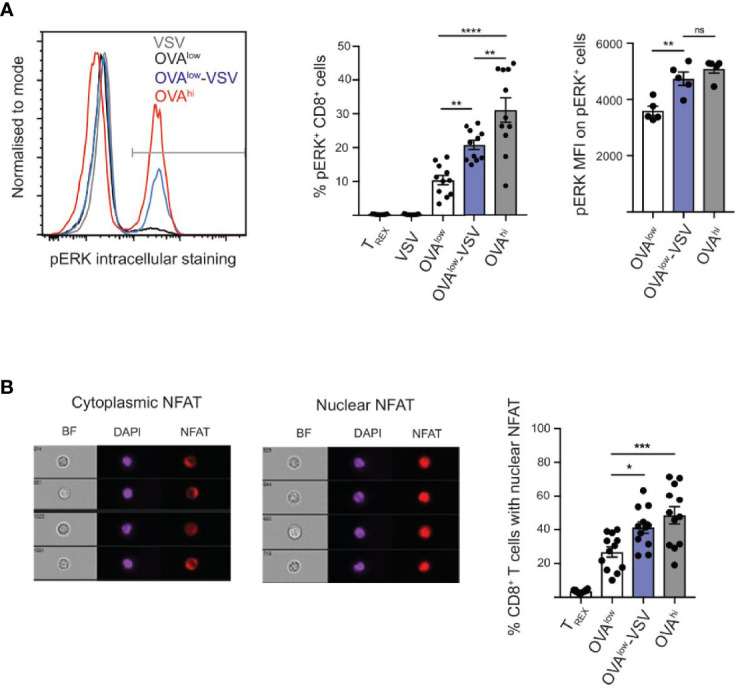
Co-agonism enhances ERK phosphorylation and NFAT nuclear translocation. *Ex vivo* OT-I lymphocytes were stimulated with the indicated CHO APCs for 3h **(A)** or 6h **(B)**, followed by fixation and intracellular staining to detect pERK **(A)** or NFAT **(B)**, and analysis using flow cytometry **(A)** or imaging flow cytometry **(B)**. **(A)** Data from 10 mice from 3 independent experiments (%) or 4 mice from 1 experiment, representative of 3 experiments (MFI). **(B)** Imaging flow cytometry images showing representative CD8^+^ T cells with cytoplasmic and nuclear NFAT. Data from 12 mice. Statistical significance was calculated using one-way ANOVA, with Tukey’s multiple comparisons test. *P < 0.05, **P < 0.01, ***P < 0.001, ****P < 0.0001, ns, P ≥ 0.05.

We have shown here that co-agonism enhances CD8^+^ T cell proliferation *in vitro* and *in vivo* (**Figure 2**), as well as CTL differentiation (**Figure 4**). We have also shown that co-agonism enhances the PI3K-Akt signaling pathway through cell-intrinsic and extrinsic modes (**Figure 3**). We then asked if co-agonism can affect some of the main components of the canonical TCR signaling pathways: ERK and NFAT. Using flow cytometry, we observed ERK phosphorylation in approximately 10% of CD8^+^ OT-I cells stimulated with OVA^low^ cells, and this proportion was doubled for OT-I cells stimulated with OVA^low^-VSV cells (**Figure 5A**), indicating that co-agonism enhances the proportion of CD8^+^ T cells that phosphorylate ERK in response to low amounts of antigen. Moreover, co-agonism enhanced the intensity (MFI) of pERK signal within the pERK^+^ population (**Figure 5A**), indicating that co-agonism increases the magnitude of pERK signaling at the single cell level within the responding population. We then used imaging flow cytometry to test whether co-agonism-aided T cell activation can enhance NFAT nuclear translocation. NFAT nuclear translocation was dependent on signal strength, with OVA^hi^ stimulation resulting in significantly higher percentage of cells with translocated NFAT as compared to OVA^low^ stimulation. Importantly, stimulation with OVA^low^-VSV cells resulted in significantly higher NFAT nuclear translocation than OVA^low^, indicating that presence of non-stimulatory pMHC enhances NFAT nuclear translocation in response to low amount of antigen (**Figure 5B**). We also investigated NFκB nuclear translocation using imaging flow cytometry, but were not able to assess the effect of co-agonism due to the very limited dynamic range of our assay (**Supplementary Figure 1E**). Our data show that co-agonism modulates T cell signaling pathways, enhancing ERK phosphorylation and NFAT nuclear translocation.

### Non-Stimulatory pMHC Promote Recruitment of Coreceptor-Bound Lck to the Immune Synapse

The presence of co-agonist pMHC class I complexes has been shown to modulate immune synapse formation, enhancing CD8 recruitment and its interaction with CD3 (7), as well as enhancing recruitment of total and active forms of Lck (9). This led to the hypothesis that coreceptor binding to co-agonist pMHC recruits coreceptor-bound Lck to the immune synapse, and that this Lck can then further phosphorylate TCR-CD3 complexes engaged by agonist pMHC complexes (4, 9). However, as only approximately 10-25% of CD8 coreceptors are associated with Lck (43–45) (**Supplementary Figure 2A**), and free Lck can also contribute to initiation of TCR signaling (45, 46), we sought to investigate the roles of coreceptor-bound and free Lck at the immune synapse formed during recognition of co-agonist pMHC complexes.

**Figure 6:**
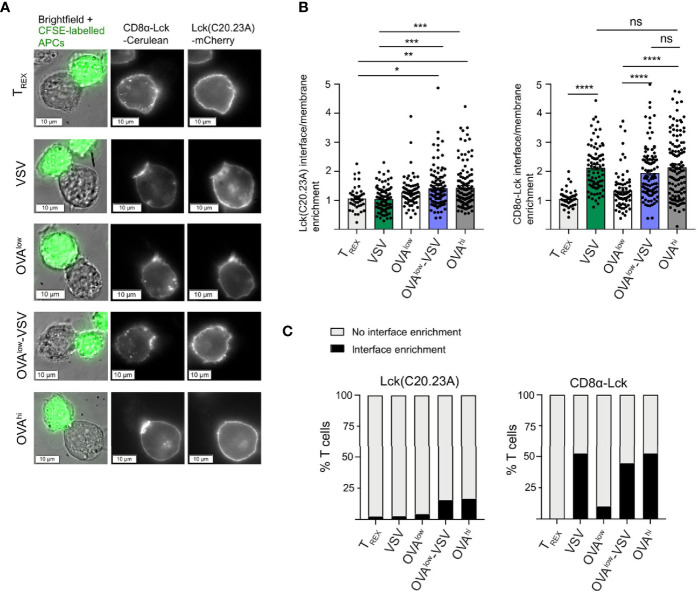
Non-stimulatory pMHC-I recruit CD8-bound Lck to the T cell: APC interface even in absence of antigenic pMHC-I. Endogenous Lck^–/–^ OT-I hybridomas co-expressing Lck(C20.23A)-mCherry (free Lck) and CD8α-Lck-Cerulean (bound Lck) were co-cultured with the CFSE-labelled CHO APCs labelled for 30 min, followed by fixation and fluorescence microscopy analysis. **(A)** Representative images of T cell: CHO APC conjugates. **(B)** Recruitment of Lck(C20.23A)-mCherry (left) and CD8α-Lck-Cerulean (right) to the T cell: APC interface. The interface recruitment was calculated as (mean pixel intensity at the interface – background)/(mean pixel intensity at membrane outside the interface – background). **(C)** Percentage of T cells with interface enrichment of free (left) and bound (right) Lck. The interface Lck enrichment was defined by the interface enrichment values of 2 and above. Data from 3 independent experiments, with 41 conjugates for T_REX_, 79 conjugates for VSV, 69 conjugates for OVA^low^, 103 conjugates for OVA^low^+VSV and 120 conjugates for OVA^hi^. Statistical significance was calculated using one-way ANOVA, with Tukey’s multiple comparisons test. *P < 0.05, **P < 0.01, ***P < 0.001, ****P < 0.0001, ns, P ≥ 0.05.

In order to investigate the recruitment of CD8-bound Lck and free Lck, we used an OT-I T cell hybridoma deficient in endogenous Lck and co-expressing fluorescent protein fusions of free and bound Lck mutants (45). Lck(C20.23A)-mCherry is unable to bind to coreceptors due to mutation of the critical di-cysteine C20.23 motif (47), whereas the CD8αLck-Cerulean construct consists of Lck covalently fused to CD8α, resulting in constitutively bound Lck (46). We co-incubated the OT-I hybridoma cells with the CFSE-labelled CHO APC panel for 30min, followed by fluorescence microscopic analysis of Lck recruitment to the T cell: APC interface (**Figure 6A**). We observed a small but statistically significant increase in recruitment of free Lck in OT-I cells interacting with OVA^hi^ or OVA^low^-VSV, as compared to OT-I cells interacting with non-stimulatory T_REX_ or VSV APCs (**Figure 6B**). Overall, synaptic recruitment of free Lck was low for all the APCs tested, with only approximately 15% of T cells interacting with OVA^hi^ or OVA^low^-VSV showing synaptic enrichment of free Lck (**Figure 6C**). On the other hand, we observed synaptic enrichment of bound Lck in approximately 50% of T cells interacting with OVA^hi^ or OVA^low^-VSV APCs, as compared to approximately 10% of T cells interacting with OVA^low^ APCs (**Figure 6B**). The role of co-agonist pMHC in mediating synaptic recruitment of coreceptor-bound Lck is further underscored by an increase in mean synaptic enrichment of CD8-Lck in T cells interacting with OVA^low^-VSV, as compared to OVA^low^ APCs (**Figure 6B**). Unexpectedly, we discovered that the presence of non-stimulatory pMHC even in the absence of agonist pMHC is sufficient to recruit CD8-bound Lck to the synapse. T cells interacting with VSV APC showed bound Lck synapse enrichment comparable to those interacting with OVA^hi^ or OVA^low^-VSV in terms of mean synaptic enrichment (**Figure 6B**) and percentage of T cells with bound Lck synapse recruitment (**Figure 6C**). These results indicate that the total amount of pMHC-I on APCs, rather than the amount of agonist pMHC-I, determines recruitment of coreceptor-bound Lck to the T cell: APC interface. It must be noted that our analysis does not provide spatial and temporal resolution to evaluate interactions between agonist and co-agonist pMHC, CD8, and Lck. Moreover, our analysis does not distinguish between active (phosphorylated at Y394) and inactive (Y394 non-phosphorylated) forms of Lck (48). To address this shortcoming, we measured CD8 association with total and active Lck using flow-IP (49, 50). This analysis showed that approximately 10% of CD8αβ coreceptors are associated with Lck, and approximately 5% of CD8αβ coreceptors are associated with Lck phosphorylated at Y394 (**Supplementary Figure 2A**). This suggests that approximately half of CD8-bound Lck is in its active form. Moreover, we did not observe increased Lck activation upon TCR simulation (**Supplementary Figure 2B**), consistent with previous reports (48, 51). Based on these findings, we propose that non-stimulatory pMHC helps to recruit active Lck to the cell membrane.

## Discussion

Non-stimulatory self pMHC can enhance murine and human CD8^+^ T cell responses to limiting amounts of antigenic pMHC complexes (6–9, 11, 12). Given that antigenic peptides can be present at relatively low numbers in the context of a large excess of non-stimulatory self pMHC-I (23–25), it seems likely that co-agonism is the main modality of TCR recognition during T cell activation under physiological conditions. We show here that co-agonism enhanced effector cytokine production, *in vitro* and *in vivo* proliferation, as well as expression of transcription factors associated with CD8^+^ T cell differentiation and effector functions. Moreover, co-agonism increased T cell responses to low amount of antigen in cell-extrinsic mode, with co-agonist primed CD8^+^ T cells enhancing PI3K/Akt signaling and proliferation in neighboring CD8^+^ T cells primed with low amounts of antigenic pMHC. Co-agonism enhanced ERK phosphorylation and NFAT nuclear translocation. Co-agonist pMHC class I complexes recruit CD8-bound Lck to the immune synapse, even in the absence of agonist pMHC.

Priming with antigen programs CD8^+^ T cells to proliferate and differentiate into effector CTLs in an antigen-independent manner (32, 52). Previous research on co-agonism in CD8^+^ T cell activation focused mainly on short term *in vitro* activation assays and did not address the role of co-agonism in programming T cell proliferation and effector functions. This was largely due to the technical difficulties in selectively controlling the amounts of agonist and non-stimulatory pMHC under physiological settings. To overcome this problem, we used an adherent xenogeneic cell line expressing agonist or non-stimulatory peptide MHC-I in a single chain format (6, 26). Using this system, we confirmed a previous report (32) that short (4h) antigen stimulation is sufficient for subsequent antigen-independent CD8^+^ T cell proliferation and effector differentiation *in vitro*. The proportion of proliferating cells was dependent on the amount of antigenic pMHC, and the presence of co-agonist pMHC-I increased the fraction of cells proliferating in response to low amounts of agonist. Moreover, the presence of co-agonist pMHC-I boosted CD8^+^ T cell effector differentiation *in vitro*. Priming for 4h with a low amount of antigen in the presence of co-agonist pMHC was sufficient to improve CD8^+^ T cell proliferation in response to bacterial infection, indicating that co-agonism boosts T cell responses *in vivo*.

Protection against pathogens requires the coordinated response of multiple immune cells. The T cell response at the system level depends on the activation status of individual T cells, but also on the individual cells sensing and responding to the abundance and activation status of other T cells in the population. There is evidence that T cell proliferation at the population level is regulated by a quorum sensing mechanism, with a critical role for autocrine IL-2 production (38, 53). IL-2 has been shown to synergize with weak TCR signals resulting from low abundance or low affinity of agonist pMHC-I to activate PI3K/Akt pathways and drive CD8^+^ T cell proliferation (38, 39). We show here that the presence of co-agonist-primed CD8^+^ T cells enhances PI3K/Akt signals and proliferation in CD8^+^ T cells stimulated by low amounts of antigen. This suggests that co-agonism regulates system-level CD8^+^ T cell responses though cell-intrinsic and extrinsic modes of action. Our current analysis does not allow us to conclude if co-agonism enhances CD8^+^ T cell quorum sensing by contact-dependent or -independent mechanisms. T cell quorum sensing is regulated by ICAM-1 mediated T cell: T cell clustering, and by interactions between CD28 and CTLA-4 with CD80/CD86 expressing T cells (54, 55). It will be of interest to determine if co-agonism augments quorum sensing through modulation of T cell: T cell clusters formation, as well as CD80 and CD86 expression on T cells, in addition to enhancing IL-2 production.

We have previously shown that co-agonism in human CD8^+^ T cells enhances proximal TCR signaling by increasing recruitment of active Lck at the immune synapse and by increasing phosphorylation of CD3ζ molecules from TCR-CD3 complex triggered by agonist pMHC (9). We therefore decided to test the effect of co-agonism on some of the main TCR signaling cascades that lead to activation of different transcription factors – ERK-MAPK pathway and NFAT pathway. Here, we show that co-agonism alters T cell signaling pathways, by enhancing ERK phosphorylation at both population and single cell level, and by increasing the proportion of cells that undergo NFAT nuclear translocation. It remains to be determined if this enhancement of T cell signaling pathways and activation by co-agonist pMHC is influenced by co-stimulation, especially in the context of priming by professional APCs. The surrogate APC system used in our experiments provides only weak co-stimulatory signals, in the form of hamster ICAM-1 expressed on CHO cells (56). As the data presented here and in our previous work (9) show that co-agonist pMHC enhance canonical TCR signaling pathways, we hypothesize co-agonism that augments TCR signal, but is distinct from and cannot substitute for co-stimulatory signal.

Unlike TCR, CD8 coreceptor binding to pMHC is independent of the sequence of the presented peptide. Therefore, CD8 coreceptor binds to agonist and co-agonist pMHC with similar affinities. Under physiological conditions of low antigen and high non-stimulatory pMHC density, CD8 binding to non-stimulatory pMHC complexes is predicted to be the main interaction involving the coreceptor at the immune synapse. Non-stimulatory pMHC have been shown to recruit CD8 to the immune synapse, and to enhance CD8-CD3 interaction (7). Consequently, CD8 binding to non-stimulatory, but not necessarily to agonist pMHC is required for co-agonism (6, 9). Moreover, CD8 binding to non-stimulatory pMHC can rescue the T cell activation defect resulting from abolishing CD8 binding to agonist pMHC (6, 9). However, the precise molecular role of CD8 binding to co-agonist pMHC is not known. Only a small fraction (estimated to be 10-25%) of CD8 molecules are bound to the kinase Lck in peripheral CD8^+^ T cells (43–45), and our flow-IP analysis shows that approximately half of CD8-bound Lck molecules are in the active form. Although CD8-Lck coupling changes during T cell development, it is not altered by T cell activation (20, 44). Moreover, Lck itself is present both in coreceptor-bound and free form in peripheral CD8^+^ T cells (45, 57), and TCR signaling can be initiated by free Lck (46). We have previously shown that co-agonist pMHC complexes recruit active Lck to the immune synapse (9), and here we show that non-stimulatory pMHC class I complexes recruit CD8-bound Lck to the immune synapse. This is independent of presence of agonist pMHC, as we observed comparable recruitment of CD8-bound Lck to contact interfaces with APCs presenting high quantities of non-stimulatory or agonist pMHC-I. This result strongly suggests that the molecular mechanism of co-agonist aided CD8^+^ T cell activation involves recruitment of CD8-bound Lck to the immune synapse through CD8 interaction with co-agonist pMHC class I. The CD8-bound Lck molecules recruited through co-agonist pMHC binding can then phosphorylate TCR-CD3 complexes at the immune synapse, resulting in enhanced T cell signaling and activation. However, our data indicate that Lck recruitment to the immune synapse is not sufficient for T cell activation. This is in agreement in with a recent report by Connolly at al (58)., which shows that local changes in plasma membrane electrostatic potential facilitate dissociation of cytoplasmic domains of bystander TCR/CD3 complexes from the plasma membrane to enhance T cell signaling and activation. This process is mediated through Ca^2+-^dependent activation of phosphatidylserine scramblase TMEM16F. Taken together with our data, this suggests a two-step model of activation enhancement by co-agonist pMHC. First, Ca^2+^ signal induced by agonist pMHC activates TMEM16F, leading to dissociation of cytoplasmic regions of TCR/CD3 from plasma membrane. The exposed CD3 ITAMs are then phosphorylated by active Lck recruited to the immune synapse through co-receptor binding to non-stimulatory pMHC.

## Materials and Methods

### Antibodies

The following antibodies were obtained from eBioscience or BD: CD8α (clone 53-6.7), CD69 (clone H1.2F3; eBioscience), IL-2 (clone JES6-5H4), TNF (clone MP6-XT22), IFN-γ (clone XMG1.2), T-bet (clone 4B10), Eomes (clone Dan11mag), CD45.1 (clone A20), CD45.2 (clone 104), H-2K^b^ (clone AF6-88.5.5.3), H-2K^b^-OVA (clone 25-D1.16) and CD107a (clone eBioH4A3). The following antibodies were obtained from Cell Signaling: c-Myc (clone D3N8F), pS6 (ribosomal protein S235/236; clone D57.2.2E), pERK (p44/42 MAPK Thr202/Tyr204; clone 197G2), NFAT (clone D43B1), NF-κB (clone D14E12), pY416 Src (clone D49G4) and pY505 Lck (#2751). Lck (clone 3A5) antibody was obtained from Santa Cruz. Secondary anti-rabbit antibodies were obtained from Invitrogen (catalogue no A-11070 and A-21245).

### Cell Culture

The generation of the CHO cell APC panel was described previously (6). The CHO cells were cultured in Ham’s F12 media (Gibco) supplemented with 5% (vol/vol) foetal bovine serum (Hyclone), penicillin/streptomycin (Hyclone) and 10 µg/ml blasticidin (Invitrogen). 0.3 mg/ml hygromycin (Invitrogen) and 0.8 mg/ml G418 (Invitrogen) was used to maintain expression of scK^b^OVA and scK^b^VSV, respectively. The CHO cells were passaged every 3-4 days using trypsin. scK^b^OVA and total H-2K^b^ surface expression on the CHO panel was regularly checked by flow cytometry. 50 ng/ml doxycycline was used to induce high scK^b^OVA expression.

The generation of Lck KO OT-I T cell hybridoma cells was described previously (45). The OT-I hybridoma cells were cultured in IMDM (Hyclone) supplemented with 10% (v/v) foetal bovine serum (Hyclone), penicillin/streptomycin (Hyclone) and 50μM β-mercaptoethanol (Sigma-Aldrich). OT-I hybridoma cells were passaged daily. Surface expression of Vα2, Vβ5, CD3ϵ, CD8α, CD8β as well as fluorescence of the Lck fusions was regularly checked by flow cytometry.

### Mice

OT-I (C57BL/6-Tg(TcraTcrb)1100Mjb/J, Jackson strain no: 003831) and CD45.1 (B6.SJL-Ptprca Pepcb/BoyJ; Jackson strain: 002014) mice were obtained from Jackson laboratories. The mice were bred and maintained under restricted flora conditions in Comparative Medicine vivarium at the National University of Singapore. Experiments were performed on 6- to 14-week-old male and female mice. All animal experiments were performed according to guidelines of the Institutional Animal Care and Use Committee at the National University of Singapore, and all animal protocols were also approved by this committee.

### T Cell Activation Experiments

CHO APCs preparation for stimulation experiments was described before (26). Briefly, one day before the T cell activation experiments, CHO cells were seeded into 96 U-bottom (for flow cytometry analysis) or 12 well flat bottom plates (for imaging flow cytometry analysis and priming for *Listeria monocytogenes* infection), at 20,000 cells in 100μl or 200,000 cells in 1ml, respectively. Plates were incubated at 37°C, 5% CO_2_ overnight. For any experiments involving transfer of T cells into APC-free wells, the CHO cells were fixed with 4% paraformaldehyde at room temperature for 20 minutes, followed by five washes with PBS. This was to prevent transfer of CHO APCs with capacity to proliferate into APC-free wells.

Complete RPMI, consisting of RPMI (HyClone) supplemented with 10% (v/v) foetal bovine serum (Hyclone), penicillin/streptomycin (Hyclone) and 50μM β-mercaptoethanol (Sigma-Aldrich) was used for all T cell activation experiments. On the day of the experiment, lymph nodes (inguinal, axillary and brachial) were extracted from mice, and placed into a 6-well plate with 5ml of complete RPMI. Lymphocyte suspensions were prepared by mashing the lymph nodes against a 70μm nylon mesh cell strainer using a 1ml syringe plunger. For analysis of cell proliferation, the lymphocytes were labelled with Cell Trace Violet or Cell Trace Far Red (Invitrogen), according to manufacturer’s instructions. Lymphocytes were counted using a Z1 particle counter (Beckman Coulter). The cell concentration was adjusted to 2x10^6^ cells/ml, and 100μl (quorum sensing experiments), 200μl (all other 96 cell plate experiments) or 1ml (12 well experiments) of T cell suspension/well was added to the APC plates. Where indicated, 1ng/ml IL-7 (Peprotech) was added to maintain cell viability. For cell transfer experiments, the lymphocytes were transferred to new, APC-free U-bottom 96 well plates at the indicated time points. For cytokine production experiments, lymphocytes were incubated with the APCs in the presence of GolgiPlug (BD Biosciences). CD107a degranulation was measured as described previously (9). H-2K^b^-OVA tetramer stimulation and analysis of Lck phosphorylation by Western blotting were performed as described previously (45, 59).

### Listeria Monocytogenes Infection

2x10^6^ OT-I lymphocytes (CD45.2^+^) primed with the indicated CHO APCs were adoptively transferred into CD45.1 recipients using retroorbital intravenous injection. The next day, the mice were infected with 10^4^ colony-forming units of LM-OVA (37). Four days later, the proportion of donor-derived CD45.2^+^ CD8^+^ T cells in the spleen was analyzed using flow cytometry.

### Flow Cytometry Analysis

Live/dead labelling was performed using a LIVE/DEAD Fixable Near-IR Dead Cell Stain kit (Invitrogen). The cell-surface antibody staining was performed on ice for 30 min, followed by one wash. For intracellular cytokine staining, intracellular fixation & permeabilization buffer set (eBioscience) was used according to manufacturer’s instructions. Foxp3/transcription factor staining buffer set (eBioscience) was used according to manufacturer’s instructions to stain for transcription factors. The paraformaldehyde fixation-methanol permeabilization for phospho-protein, c-Myc and NFAT staining was described in detail before (60); this protocol was used for both flow cytometry and imaging flow cytometry analysis. Flow cytometry analysis was performed on a Fortessa X-20 (BD Biosciences), with FACSDiva used for acquisition and FlowJo versions 9 and 10 used for analysis.

### Imaging Flow Cytometry

Samples were analyzed using Amnis^®^ ImageStream^®^X Mk II Imaging Flow Cytometer (Luminex Corporation, Texas, USA). The data were analyzed using IDEAS software (Luminex Corporation, Texas, USA). For analysis of NFAT nuclear translocation, IDEAS Nuclear Localization Wizard was used.

### Microscopy Analysis of Lck Recruitment to the Synapse

CHO cells were labelled with 0.5 μM CellTrace CFSE (Invitrogen) prior to the co-incubation with OT-I hybridoma cells, to facilitate identification of APCs (CFSE^+^) and OT-I hybridoma cells (CFSE^-^) within conjugates. The labelled CHO cells and OT-I hybridomas were co-cultured in Lab-Tek II 8-well glass chambers (Nunc) for 30 minutes at 37°C, with 50,000 cells CHO cells and 50,000 OT-I hybridoma cells/well. Samples were fixed at 4% paraformaldehyde (Thermo Scientific) for 30 minutes and subsequently washed with PBS. The samples were then analyzed using Olympus IX83 microscope (Olympus Corporation) under widefield mode. The images were analyzed using cellSens Dimension software (Olympus Corporation).

### Flow-IP

Flow cytometry immunoprecipitation (FC-IP) was performed as described previously (45, 49, 50). Briefly, anti-CD8β (clone eBioH35-17.2), anti-CD8α (clone 53-6.7, BD Pharmingen), anti-H2K^b^ as negative control for background subtraction (AF6-88.5, BioLegend) were coated onto CML beads (Invitrogen, C37255). The coated beads were incubated overnight at 4°C with cell lysates from 2 x 10^6^ T cells, with 2.5 x 10^5^ capture beads used for 200 μl lysate. Beads were then washed and split equally for different staining regimes. PE-conjugated, anti-CD8α (clone 53-6.7, BD Pharmingen), anti-CD8β (clone BioH35-17.2, eBioscience) anti-Lck (clone 3A5, Santa Cruz) antibodies were used to quantify CD8/Lck coupling ratios. Anti-Src-pY416 antibody (which reacts with Lck pY394) was obtained from Cell Signaling, and detected with PE conjugated monoclonal anti-rabbit antibody (Abcam). The PE conjugated antibodies were purified with Superdex 200 10/300 GL size exclusion column for antibody:PE = 1:1 ratio if required. Calibration beads (BD Quantibrate beads, 340495) were used the number of PE molecules. Percentages of CD8 bound to Lck were calculated using the following equation: (number of Lck molecules/CD8β IP bead – Lck signal/H-2K^b^ IP bead)/(number of CD8α molecules/CD8β IP bead - CD8α signal/H-2K^b^ IP bead).

## Data Availability Statement

The raw data supporting the conclusions of this article will be made available by the authors, without undue reservation.

## Author Contributions

XZ, L-ZW, EN, KL, QW, and JB performed the experiments. XZ, L-ZW, EN, and JB analyzed the data. JB and NG designed the study and wrote the manuscript. All authors discussed the results and commended on the manuscript. All authors contributed to the article and approved the submitted version.

## Funding

This research was supported by the Singapore Ministry of Health’s National Medical Research Council under its CBRG/0064/2014 and OFIRG19nov-0066 to NG.

## Conflict of Interest

The authors declare that the research was conducted in the absence of any commercial or financial relationships that could be construed as a potential conflict of interest.

## Publisher’s Note

All claims expressed in this article are solely those of the authors and do not necessarily represent those of their affiliated organizations, or those of the publisher, the editors and the reviewers. Any product that may be evaluated in this article, or claim that may be made by its manufacturer, is not guaranteed or endorsed by the publisher.

## Supplementary Material

Supplementary Figure 1 Co-agonism enhances CD8 T cell responses. **(A)** Representative H-2K^b^ and H-2K^b^-OVA staining of antigen presenting cells used. MFI values are shown next to flow cytometry plots. **(B)** Time course of CD69 upregulation in response to stimulation with the indicated CHO APCs. Data from 4 mice from 1 experiment, representative of 2 experiments. **(C)** CD69 upregulation after 3h stimulation with the indicated CHO APCs. Data from 4 mice from 1 experiment, representative of 2 experiments. **(D)** *Ex vivo* OT-I lymphocytes were stimulated with the indicated CHO APCs for 4h, cultured for 3 days without APCs, followed by 6h re-stimulation with OVA^hi^ APCs. Degranulation of CD8^+^ T cells was quantified using CD107a staining. Data from 4 mice from 1 experiment, representative of 2 experiments. Statistical significance was calculated using t-test. *P < 0.05. **(E)** NFκB nuclear translocation after 6h stimulation with the indicated APCs. Data pooled from 6 mice from 2 experiments, analyzed using one-way ANOVA. ***P < 0.001, ns, P ≥ 0.05. Representative images show translocated and non-translocated NF-κB.

Supplementary Figure 2 Assessing Lck phosphorylation. **(A)** Quantification of CD8 and Lck coupling using flow-IP. Data pooled from 2 experiments. **(B)** Ex vivo OTI CD8^+^ T cells were stimulated using H-2K^b^-OVA tetramers for the indicated times. Lck phosphorylation was assessed using Western blotting. Data from 1 experiment, representative of 2 experiments.
