# Supplementary figures and images for: Non-Stimulatory pMHC Enhance CD8 T Cell Effector Functions by Recruiting Coreceptor-Bound Lck
Source: Front Immunol. 2021 Oct 11;12:721722. doi: 10.3389/fimmu.2021.721722 (PMC8542885; doi:10.3389/fimmu.2021.721722)

**A**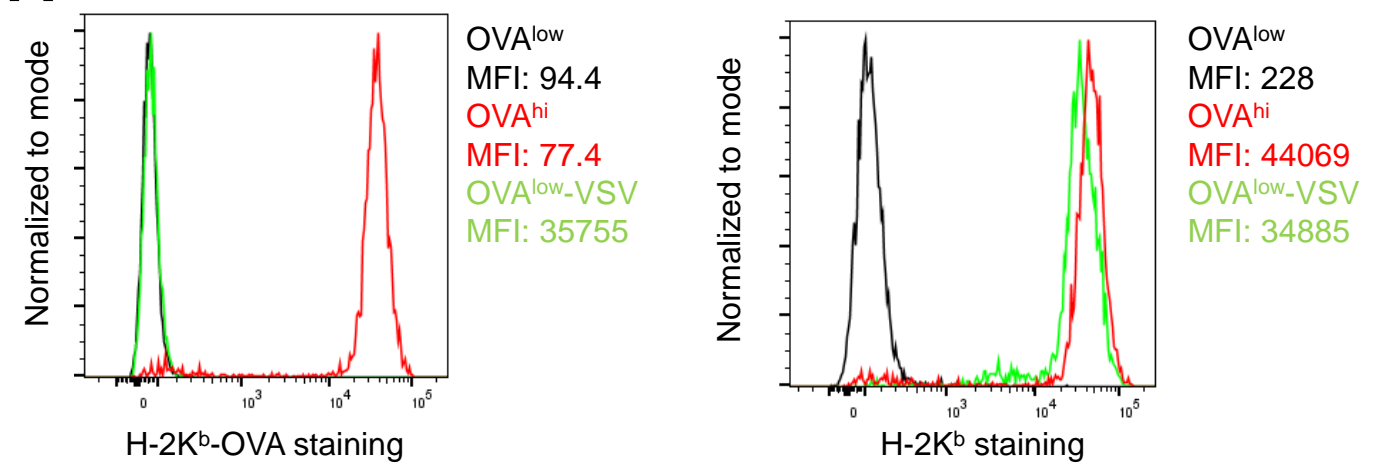**B**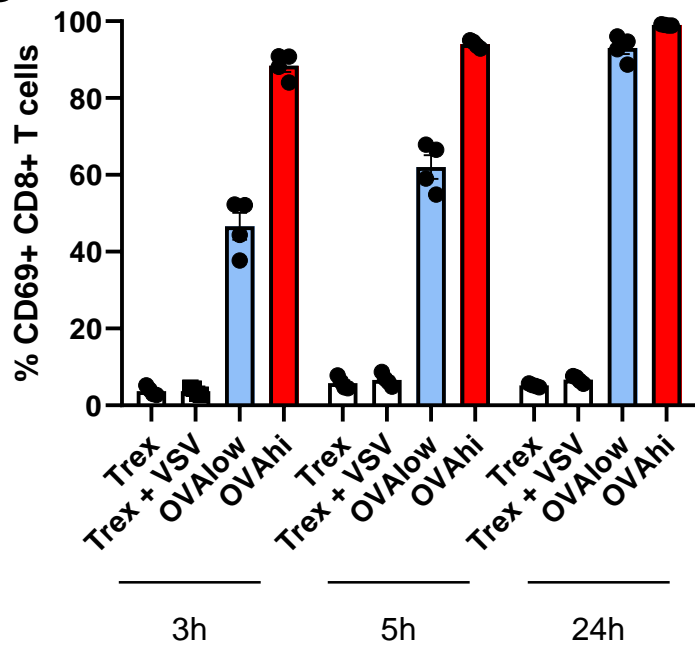**C**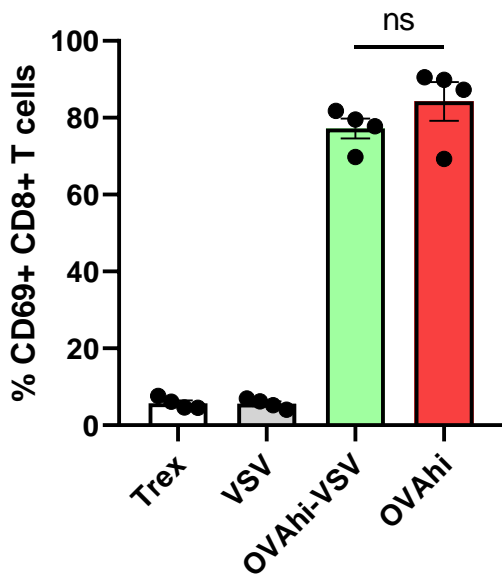**D**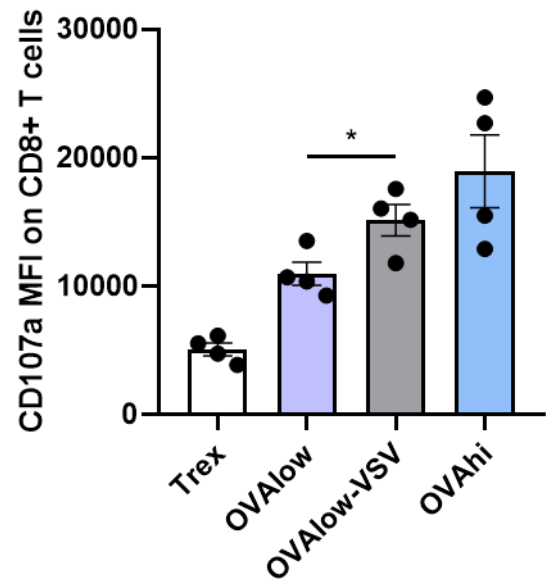**E**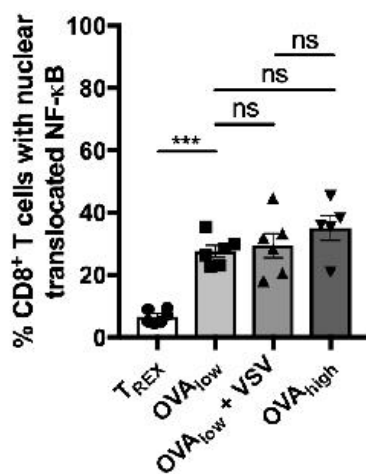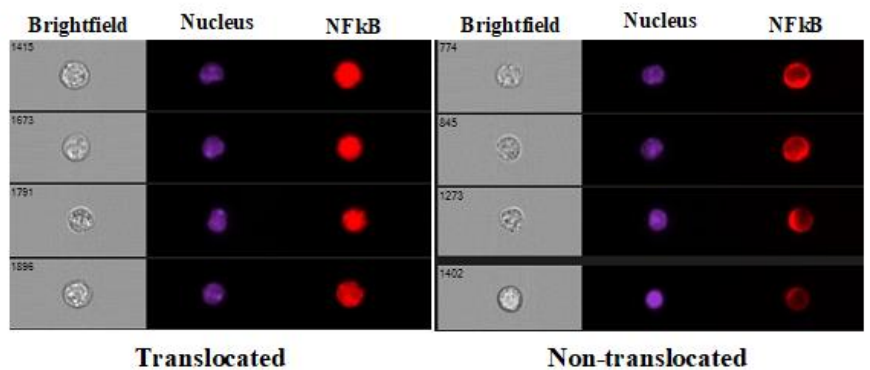

**A**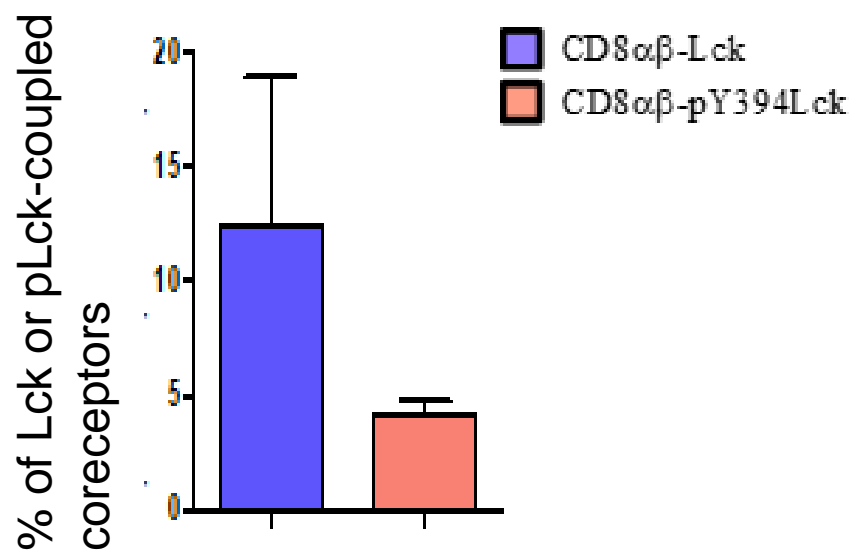**B**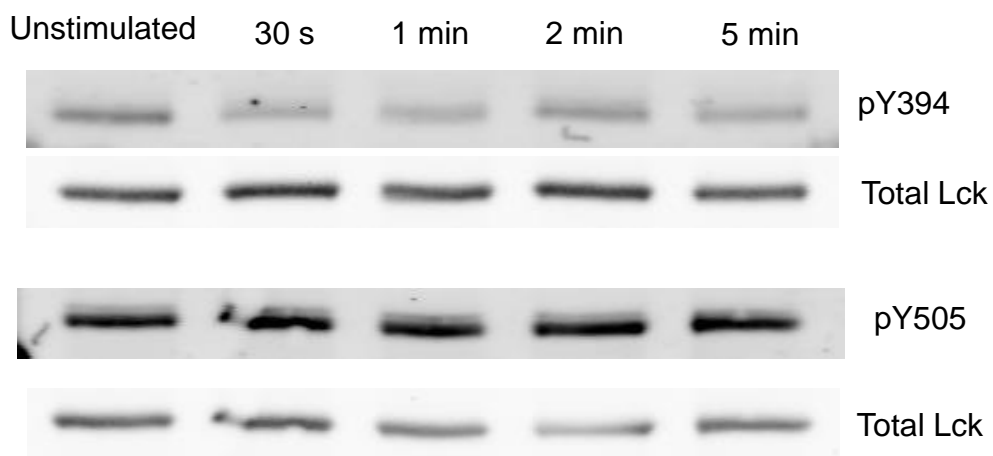

Supplement: Supplementary Figure 1 — Co-agonism enhances CD8 T cell responses. (A) Representative H-2Kb and H-2Kb-OVA staining of antigen presenting cells used. MFI values are shown next to flow cytometry plots. (B) Time course of CD69 upregulation in response to stimulation with the indicated CHO APCs. Data from 4 mice from 1 experiment, representative of 2 experiments. (C) CD69 upregulation after 3h stimulation with the indicated CHO APCs. Data from 4 mice from 1 experiment, representative of 2 experiments. (D) Ex vivo OT-I lymphocytes were stimulated with the indicated CHO APCs for 4h, cultured for 3 days without APCs, followed by 6h re-stimulation with OVAhi APCs. Degranulation of CD8+ T cells was quantified using CD107a staining. Data from 4 mice from 1 experiment, representative of 2 experiments. Statistical significance was calculated using t-test. *P < 0.05. (E) NFκB nuclear translocation after 6h stimulation with the indicated APCs. Data pooled from 6 mice from 2 experiments, analyzed using one-way ANOVA. ***P < 0.001, ns, P ≥ 0.05. Representative images show translocated and non-translocated NF-κB. [file DataSheet_1.pdf]
